# A cross-sectional survey of men’s attitudes towards public health services and their utilisation practices in Limpopo Province, South Africa

**DOI:** 10.4102/safp.v68i1.6245

**Published:** 2026-05-19

**Authors:** Lazarros Chavalala, Lufuno Makhado, Rachel T. Lebese

**Affiliations:** 1 Department of Public Health, Faculty of Health Sciences, University of Venda, Thohoyandou, South Africa

**Keywords:** attitudes, health, healthcare services, men, practices

## Abstract

**Background:**

The attitude that one holds towards healthcare services is an important factor associated with health-seeking behaviour. People with bad experiences of previously used healthcare services are less likely to engage with healthcare services; the healthcare worker-patient relationship is an important factor influencing attitudes towards healthcare services. This study aimed to determine the attitude of men towards public health services and utilisation practices.

**Methods:**

A self-administered questionnaire was administered using a quantitative cross-sectional design on 400 men conveniently selected from randomly selected communities within the selected districts. Data from 387 questionnaires successfully returned were analysed using the Statistical Package for Social Sciences version (SPSS) 29.0. for Windows.

**Results:**

Out of 387 men, 310 (81%) reported that during consultation, healthcare professionals do not respect patients, and the majority (88%; 339) reported that the waiting period in public healthcare facilities is very long. Most men (84%, 322) felt more at ease going to a private health facility than a public one. About 54% (204) of the men visited public health facilities to seek health services. There was a statistically significant relationship between men’s level of education and utilisation of healthcare services (*p* = 0.005).

**Conclusion:**

These men held negative attitude towards public health facilities and reported a lack of respect for patients by health workers. Revitalisation of work ethics and professional conduct among public healthcare workers is needed to remind workers about their code of conduct and improve the provision of public health services in a manner that users feel comfortable visiting health facilities to seek the services.

**Contribution:**

The study brings meaningful insights of men’s attitudes towards public health services and may help to better understand why few men engage in health services.

## Introduction

In many communities, visiting health facilities to seek healthcare was seen as necessary for women and children only.^1^ Moreover, clinics are often perceived as ‘female spaces’, as females usually make up most of the patients and caregivers’ population in many settings.^2^ It is of paramount importance to find out about the attitudes men hold towards health services, especially public health services. This is because even though public health services are provided at no cost in most countries, men engage with the services at low rates. Research^3^ indicates that compared to women, men have lower life expectancies and higher death rates because they are less likely to get tested for HIV, start antiretroviral treatment (ART), start ART with advanced HIV illness, and do so at older ages. They also remain undiagnosed for HIV because of the underutilisation of health services.^4^ However, men typically visit the doctor half as frequently as women do in their early adult years.^5^ It is concerning that men in sub-Saharan Africa continue to fall behind women in health services utilisation, despite the geographical, financial and cultural accessibility of community healthcare centres.^6^ Additionally, they are proportionally less likely to test for HIV and begin treatment regimens and are more likely to die while on treatment.^7^ Globally, men accounted for 52% of all deaths from non-communicable diseases (NCDs) in 2012 and were more likely than females to die prematurely from NCDs in every country.^7^ Men who visit health facilities do so as a last resort.^8^ In recent years, health research has also shifted focus to men’s health because of men experiencing poor health outcomes. However, studies conducted by Peate^9^ and Hall^10^ focused on men’s attitudes toward health issues and help-seeking rather than men’s attitudes towards public health services. Within the study setting, there are no known studies conducted to determine men’s attitudes towards public health services. This study aimed to determine men’s attitudes towards public health services and utilisation practices in Limpopo Province to gain an understanding of how men think about public health services and interact with the services. In this study, the researchers report on 387 men who completed distributed self-administered questionnaires to determine attitudes and public health services utilisation practices.

## Research methods and design

The methods used in this study are the same as those described in the study of Chavalala et al.^11^ as presented in the study design below. Although this study used similar methods, the two studies measured different variables.

### Study design

A quantitative cross-sectional design was used to determine the men’s attitudes towards public health services and utilisation practices in the Mopani, Vhembe and Capricorn districts of Limpopo province.

### Study setting

There are 454 clinics, 26 community health centres, and 41 hospitals in the province of Limpopo, some of which are district, regional, tertiary and speciality hospitals.^12^ The majority of people in Limpopo take between 15 min – 29 min to arrive at the nearest medical health facility. The majority of residents walk to the nearest medical health facilities, followed by those using public transport to get there.^13^ For many years, traditional healers have been used as an alternative health treatment option. Unemployment, inequality and poverty are some of the challenges facing the province, and about 17.6% of the population was illiterate in 2019.^14^

### Sampling

Four hundred men were conveniently sampled from five villages randomly selected in Mopani, Vhembe and Capricorn districts. Convenience sampling allowed the researcher to select men who were easily available and willing to participate in selected villages or communities because of the challenges of accessing men. Random sampling ensured an equal chance of selection in all villages/communities in the above-mentioned districts. The sample size was determined using Slovin’s formula to ensure fair representation of men in the selected districts. A population estimate of 24 500 men (*N* = 24 500) and a margin of error of 5% (*e* = 0.05) were used in the calculation, yielding a minimum sample size of approximately 394 participants. The study excluded men who refused to participate and those under the age of 18 years.

### Data collection

A structured questionnaire was used to collect data for the study. The instrument was developed by the authors following an extensive review of the literature, identification of knowledge gaps and alignment with the study’s research questions. The data collection tool was validated by experts in the field to guarantee content validity and administered to 10% (40 men) of the sample size for testing to identify any problems the tool could have contained. The men used to test the data collection tool did not form part of the actual study and were from another district that was not part of the study. To accommodate respondents who were not fluent in English, the instrument was developed in English and translated into Xitsonga, Tshivenda and Sepedi by qualified language practitioners in these languages. The questionnaire had closed-ended questions about public healthcare utilisation practices, attitude towards public health services and demographic data. Researchers made appointments with selected men who voluntarily agreed to participate and met them at local churches, chiefs’ kraal and schools, depending on where they felt more comfortable completing the questionnaires in each selected community.

### Data analysis

A Statistical Package for Social Sciences (SPSS) version 29.0 was used to code the data. Data were then cleaned to ensure quality by finding errors and confirming with the sources. Respondents’ demographic characteristics, attitudes towards public health services, and utilisation practices were all described using descriptive statistics and presented in frequency tables. Inferential statistics were used to perform statistical tests to measure the association between variables contained in the study data, and all tests were two-tailed, and the significance level was set at 0.05. The chi-square test of association was used to measure the association between men’s level of education, their practices and patterns of health services utilisation.

### Ethical considerations

An application for full ethical approval was made to the University of Venda Research Ethics Committee and ethics consent was received on 15 December 2021. The ethics approval number is FHS/21/PH/26/1215. The headmen and chiefs of the sampled communities granted permission for the study to be carried out. Before taking part in the study, each male respondent freely gave written informed consent after being fully informed about the study goal and the procedure of participation.

## Results

A total of 387 of the 400 self-administered questionnaires that were distributed were correctly completed and returned, yielding a 97% response rate. The respondents’ ages ranged from 18–71 years, with an average age of 36 years. As shown in Table 1, 67% (*n* = 259) were single, and about 66% (*n* = 255) were unemployed. Most of the men (41%) lived in the Mopani district, and 44% (*n* = 170) attained tertiary education and Christianity religion was the most common religion among men (65%; *n* = 259).

**TABLE 1:** Respondents’ sociodemographic characteristics.

Characteristics	*n*	%
**Marital status (*N* = 387)**		
Single	259	67
Married	100	26
Divorced	16	4
Cohabiting	4	1
Widower	8	2
**Employment status (*N* = 380)**		
Employed	103	27
Unemployed	255	59
Self-employed	52	14
**District (*N* = 387)**		
Mopani	159	41
Vhembe	131	34
Capricorn	97	25
**Level of education (*N* = 386)**		
Never attended school	5	1
Did not finish elementary school	6	2
Primary	29	7
Did not finish high school	17	4
Secondary	138	36
Attended tertiary education but did not complete	21	5
Tertiary	170	44
**Religion (*N* = 384)**		
Christian	249	65
Islam	4	1
Hinduism	4	1
African traditional religion	127	33

## Attitude of men towards public healthcare services

As presented in Table 2, the number of responses differs from one item to the next. About 81% (*n* = 310) agreed that healthcare workers do not respect patients during consultation, while about 88% (*n* = 339) believed that the average waiting period at public health facilities is very long. The results also show that 84% (*n* = 322) felt more comfortable visiting a private medical institution for health services as opposed to a public health facility, and about 85% (*n* = 324) agreed that the queue to see a healthcare worker at public health facilities is often long and moves at a slow pace. Moreover, about 79% (*n* = 304) agreed that healthcare workers leave patients unattended without informing patients of their absence or commitment, keeping them busy, while 55% (*n* = 208) felt that the facilities they visited met their expectations. Moreover, half of the respondents (50%; *n* = 189) felt that healthcare workers were friendly and approachable, and 48% (*n* = 185) felt healthcare workers present a good attitude toward patients. Less than half (47%; *n* = 179) of respondents agreed that healthcare workers welcome patients with a smile. More than half of the respondents (51%; *n* = 192) agreed that healthcare workers give patients advice politely, and 72% (*n* = 274) agreed that healthcare workers often verbally abuse patients. The majority (84%; *n* = 322) of men agreed that healthcare workers give preferential treatment to people who are top government officials and businesspeople, as such people do not join queues even when they arrive late; they skip the queue and get attended to first.

**TABLE 2:** Attitude of men towards public healthcare services.

Statement	Agree		Not sure		Disagree		Total response
	*n*	%	*n*	%	*n*	%	
Healthcare staff do not respect patients during consultation	310	81	35	9	37	10	382
At the public health facility, the waiting period is typically quite lengthy	339	88	29	8	15	4	383
I feel more at ease going to a private health facility than a public one.	322	84	25	7	36	9	383
At the public health facility, the queue to see a health worker is often long and moves at a slow pace	324	85	47	12	12	3	383
Healthcare workers leave patients unattended, without informing them of their absence or the commitments keeping them busy	304	79	42	11	38	10	384
The health facility met my expectations during my visit	208	55	38	10	135	35	381
Health workers are friendly and approachable	189	50	42	11	149	39	380
Health workers have a good attitude towards patients	185	48	47	12	151	40	383
Health workers welcome clients with a smile	179	47	35	9	166	43	380
Health workers provide advice in a polite way	192	51	35	9	152	40	379
Health workers often verbally abuse patients	274	72	38	10	68	18	380
Patients who are senior government employees, businesspeople and traditional leaders are sometimes given preferential treatment by Health workers; such people do not join long queues even when they arrive late	322	84	45	12	14	3	381

## Men’s utilisation of public healthcare services

Over half (54%; *n* = 204) of men went to public health facilities to access health services. The findings further show that most (35%; *n* = 71) men visited public healthcare facilities more than a year ago, as shown in Table 3.

**TABLE 3:** Public healthcare facilities utilisation.

Question	*n*	%
**Have you ever visited a public health facility?**		
Yes	204	54
No	176	46
**When last did you visit a public health facility?**		
Less than a 1 month ago	44	22
Between 1–3 months ago	33	16
More than 3 months ago	20	10
More than 6 months ago	36	18
More than 1 year ago	71	35

### Association between men’s level of education and practices on health services utilisation

As presented in Table 4, 91 men with tertiary education certificates had never visited health facilities to utilise healthcare services. On the other hand, Table 4 also indicates that 86 men out of 134 respondents with secondary education visited public health facilities to seek healthcare services. Table 4 further indicates that 17 men of the 28 with primary education also visited public health facilities to seek healthcare services. However, there was a statistically significant association between respondents’ level of education and utilisation of healthcare services (*p* = 0.005).

**TABLE 4:** Cross-tabulation of level of education and health services utilisation.

What is your level of education	Have you ever visited a health facility to seek healthcare services?				Total	Pearson Chi-square *P*-value
	Yes		No			
	*n*	%	*n*	%		
Never been to school	4	80	1	20	5	0.005
Did not finish primary school	0	0	6	100	6	-
Primary	17	60	12	40	29	-
Did not finish high school	9	52	8	48	17	-
Secondary	86	64	48	36	134	-
Been to tertiary but did not finish	10	50	10	50	20	-
Tertiary	78	46	91	54	169	-

**Total**	**204**	**54**	**176**	**46**	**380**	**-**

### Association between men’s level of education and patterns of public health services utilisation

As presented in Table 5, 29 men with tertiary education visited healthcare facilities more than a year ago, while two men without any education visited health facilities less than a month ago. Table 5 also shows that 26 men with secondary education visited health facilities more than a year ago. However, there was no statistically significant association between men’s level of education and pattern of public healthcare services utilisation (*p* = 0.154).

**TABLE 5:** Crosstabulation of level of education and patterns of healthcare services utilisation.

Level of education	When did you last visit a health facility to seek healthcare services?										Total	Pearson Chi-square *P*-value
	Less than a 1 month ago		Between 1–3 months ago		More than 3 months ago		More than 6 months ago		More than 1 year ago			
	*n*	%	*n*	%	*n*	%	*n*	%	*n*	%		
Never been to school	2	50	0	0	0	0	1	25	1	25	4	0.154
Primary	4	25	4	25	1	6	4	25	3	19	16	-
Did not finish high school	2	-	2	-	0	0	1	-	4	-	9	-
Secondary	16	19	19	22	6	7	19	22	26	30	86	-
Been to tertiary but did not finish	1	10	0	0	1	10	0	0	8	80	10	-
Tertiary	19	24	8	10	12	15	11	14	29	37	79	-

**Total**	**44**	**21**	**33**	**16**	**20**	**10**	**36**	**18**	**71**	**35**	**204**	**-**

## Discussion

The study’s respondents ranged in age from 18–71 years old, with an average age of 36 years old. Men who fall within the average age in this study are likely to be sexually active. These men are likely to be infected with STIs, including HIV, if they engage in risky sexual practices such as having multiple partners and not using condoms.^15^ The majority of respondents were single. This suggests that health-seeking behaviour may be lower among most respondents. Lower engagement may be associated with a lack of social support to engage in health-seeking behaviour and challenge their attitudes towards health services, compared to those who are married. Blumberg et al.^16^ attest that married people tend to seek healthcare more than those who are not married. Witty et al.^17^ also attest that men’s utilisation of healthcare facilities is influenced by social and family support. Most respondents were unemployed, which may be associated with differences in attitudes towards public health services and utilisation patterns, potentially reflecting underlying socioeconomic barriers such as limited financial resources and challenges related to accessing care. Being unemployed is likely to affect access to health services, especially where health services are paid for or where travelling may be required using paid transport. It was encouraging that most of the respondents held tertiary qualifications. This may indicate that most respondents are literate and may find it easier to understand health-related information, which could be associated with more favourable attitudes towards health services. Moreover, the majority of respondents were Christians.

It is concerning that most respondents reported that healthcare workers do not respect patients during consultation and are verbally abusive. The observed lack of respect could be associated with respondents’ reluctance to revisit the facilities for further use and possibly perpetuate a negative attitude toward public health services. This study’s findings confirm the findings of Sithole,^18^ who discovered that many male patients complained about being disrespected by health staff and that the staff were often unpleasant and unhelpful. Additionally, the study findings of Uwimana et al.^19^ are also in support of the current study findings, as males in their study reported being disrespected by health staff at public health facilities in Rwanda. However, it is concerning that healthcare workers were viewed as verbally abusive. These findings also confirm the findings of Kassa et al.^20^ where patients reported verbal and physical abuse from nurses. Moreover, this study established that public healthcare workers give preferential treatment to top government officials, businesspeople and their friends. These findings suggest that Batho Pele’s principle, which speaks of giving patients respect, may have been overlooked, and likely, the principle is not adhered to when such behaviour occurs.

Moreover, it was noted from the findings that the average waiting time was a concern for respondents. This suggests that men may be less likely to visit public health facilities to avoid waiting longer before seeing a clinician. The National Guideline on Management of Patient Waiting Time in Clinics, Community Health Centres (CHCs), and Outpatient Departments of Public Hospitals in South Africa states that while the total time spent receiving services at various service points per patient visit is 60 min (1 h), the ideal maximum patient waiting time for services in clinics and CHCs is 120 min (2 h). Therefore, a patient’s maximum visitation period at the facility is 180 min, or 3 h.^21^ These findings highlight the need to monitor the waiting period at public health facilities to ensure compliance with the National guidelines on Waiting period at public health facilities in South Africa. The findings further highlight the need for patient education on waiting time at public health facilities to help patients understand why they are waiting for a prescribed period, as stipulated in the national guideline on management of patient waiting time in public health facilities in South Africa.

Moreover, respondents reported being more comfortable visiting a private health facility than a public one. These findings concur with the findings of Uwimana et al.,^17^ who discovered that males in their study expressed a preference for private health facilities to receive healthcare services rather than going to public health facilities, because private facilities are better in terms of staff attitude. These findings suggest that the staff attitude in public health facilities may be associated with lower utilisation of public health services by men; hence, they prefer private health service providers. Poor communication and neglect of patients were noted from the findings, as respondents reported being left unattended without health workers communicating their absence or commitment, keeping them busy. These findings are supported by the work of Mabusela and Ramukumba,^22^ who found that newly qualified nurses were accused of showing a lack of dedication to their jobs and using their phones while neglecting patients. These findings suggest that healthcare workers, particularly nurses, are likely to deviate from their professional code of conduct to respect and care for patients. Despite the negative experience that most respondents have had, it is encouraging that respondents were satisfied with the service received at the visited facilities, as healthcare workers were friendly and approachable, welcomed them with smiles, and politely gave advice. The findings highlight that within various public health facilities respondents visited, there are still healthcare workers doing their work with commitment and ensuring that patients’ needs are met. These findings concur with the findings of Haskins et al.,^23^ who discovered that patients in their survey expressed satisfaction with the nursing care and services they received at the medical institutions they visited.

It has been noted from the current study findings that respondents have visited public health facilities to seek assistance. However, the visits were not regular as the majority had visited more than 1 a year at the time of the study. Irregular men’s visits to health facilities were also noted in the study of Thorp et al.,^24^ where 82% of men who were participants had at least visited a health facility a year before the study. The current study findings indicate that men utilise public health services; however, they are not engaging with health services regularly as they should. Moreover, this study established that respondents’ level of education was associated with visiting public health facilities to seek healthcare services, as there was a statistically significant relationship between respondents’ level of education and public healthcare services utilisation (*p* = 0.005). However, respondents’ level of education was not associated with the pattern of public healthcare services utilisation, as there was no statistically significant association between respondents’ level of education and pattern of public healthcare services utilisation (*p* = 0.154).

The authors acknowledge that the use of convenience sampling may somehow limit the representativeness of the findings. This study focused on men who live in rural areas, and the findings may not be generalised to men living in townships and urban areas. The authors are of the view that men in township and urban areas may hold different perspectives on public health facilities and services offered. Future research could focus on determining the attitudes of men residing in townships and urban areas. Despite the given limitation, our analysis gave new insights and findings that are in line with previous studies. There is a need for qualitative research to explore male patients’ experiences. In practice, there is a need to implement continuous patient satisfaction surveys to identify aspects that patients are not happy with within the public health facilities. There is a need to revive work ethics and professional conduct among public healthcare workers to strengthen the healthcare worker-client relationship.

## Conclusion

The study found that men held negative attitudes towards public healthcare services and did not regularly visit public health facilities to seek healthcare. There is a need to revive work ethics and professional conduct among public health workers. Future research could explore male patients’ experiences of services received at visited public health facilities qualitatively as well.
